# Effect of drug utilization reviews on the quality of in-hospital prescribing: a quasi-experimental study

**DOI:** 10.1186/1472-6963-6-33

**Published:** 2006-03-14

**Authors:** Jean-Pierre Grégoire, Jocelyne Moisan, Louise Potvin, Isabelle Chabot, René Verreault, Alain Milot

**Affiliations:** 1 Population Health Research Unit, Centre hospitalier affilié universitaire de Québec, 1050 Chemin Ste-Foy, Québec, Qc, G1S 4L8, Canada; 2 Faculty of Pharmacy, Université Laval, Québec, Qc, Canada; 3 Groupe de recherche interdisciplinaire en santé, Université de Montréal, Montréal, Qc, H3C 3J7, Canada; 4 Geriatric Research Unit, Centre hospitalier affilié universitaire de Québec, 1050 Chemin Ste-Foy Québec, Qc, G1S 4L8, Canada; 5 Faculty of Medicine, Université Laval, Québec, Qc, Canada; 6 Centre hospitalier universitaire de Québec, 10 de l'Espinay, Québec, Qc, G1L 3L5, Canada

## Abstract

**Background:**

Drug utilization review (DUR) programs are being conducted in Canadian hospitals with the aim of improving the appropriateness of prescriptions. However, there is little evidence of their effectiveness. The objective of this study was to assess the impact of both a retrospective and a concurrent DUR programs on the quality of in-hospital prescribing.

**Methods:**

We conducted an interrupted time series quasi-experimental study. Using explicit criteria for quality of prescribing, the natural history of cisapride prescription was established retrospectively in three university-affiliated hospitals. A retrospective DUR was implemented in one of the hospitals, a concurrent DUR in another, whereas the third hospital served as a control. An archivist abstracted records of all patients who were prescribed cisapride during the observation period. The effect of DURs relative to the control hospital was determined by comparing estimated regression coefficients from the time series models and by testing the statistical significance using a 2-tailed Student's t test.

**Results:**

The concurrent DUR program significantly improved the appropriateness of prescriptions for the indication for use whereas the retrospective DUR brought about no significant effect on the quality of prescribing.

**Conclusion:**

Results suggest a retrospective DUR approach may not be sufficient to improve the quality of prescribing. However, a concurrent DUR strategy, with direct feedback to prescribers seems effective and should be tested in other settings with other drugs.

## Background

Prescription drugs constitute an important component of health care. However, drugs can only benefit to patients if they are used appropriately which involves that physicians prescribe them according to evidence. One common method of assessing and correcting the appropriateness of drug prescription is drug utilization review (DUR) programs [1-3].

In a retrospective DUR, observed patterns of drug prescriptions together with an evaluation of their appropriateness are sent to physicians in the form of a report of collated practice patterns. Since the prescriptions assessed were issued in the past, a retrospective DUR is relevant at preventing inappropriate prescribing in the future [2]. A DUR can also be run concurrently to the intervention. The appropriateness of prescriptions is evaluated after the drug has been dispensed but while the patient is still hospitalized. The patient may then benefit from any corrective action. Such action may take the form of individual feedback from a pharmacist to the physician. A concurrent DUR aims both at improving current prescribing patterns and at preventing inappropriate prescribing in the future [2].

The effectiveness of DUR programs has yet to be established. The few evaluation studies of those programs conducted until now have been criticized for lack of rigor. In general, there are no adequate control groups and prior trends in the quality of prescribing is not taken into account [2,4-6]. The present study was designed to avoid those limitations by comparing the effectiveness of retrospective and concurrent DUR to improve the quality of physician drug prescriptions in hospital settings.

## Methods

### Design

We conducted an interrupted time-series quasi-experimental study [7] in three hospitals. The natural history of the prescription was established prior to the intervention in two experimental hospitals. A retrospective DUR was then implemented in one of the hospitals, a concurrent DUR in another, whereas the third hospital served as a control. The three hospitals are independently managed from each other although they are all affiliated with Laval University. They deliver tertiary care to a population of around 750,000 inhabitants in Quebec City area.

### Selection of the drug

To remain as close as possible to the routine clinical practice setting, the targeted drug was selected after consulting pharmacists in all three settings to identify a drug that: 1) would be a clinically relevant target for a DUR, 2) had been prescribed in the hospitals for at least two years, 3) had not been subjected to any prior DUR or intervention aiming at modifying physicians prescribing behaviors, and 4) was being prescribed at least ten times per month. Cisapride was the only drug to meet these criteria in all three settings. Cisapride is a gastrointestinal tract promotility agent that was frequently used in Canada before its withdrawal from the market in 2000, after completion of this study.

### Explicit criteria

Criteria for the appropriate prescription of cisapride were made available to us by the Quebec hospitals network on DUR [8]. The criteria had been developed by experts, and approved by the network's scientific committee. We used criteria pertaining to indication for use, combination therapy, dosage, and drug interactions (Table 1). The Pharmacology and Therapeutic Committees in both the concurrent and the retrospective DUR hospitals approved those criteria.

### Interventions

#### Retrospective DUR hospital

On March 2, 1998, the Pharmacy department in the retrospective DUR hospital distributed a newsletter informing all physicians and pharmacists on the upcoming DUR on cisapride. The newsletter included criteria for the appropriate prescribing of cisapride. Using the explicit criteria, all prescriptions dispensed between March 16 and May 24, 1998 were retrospectively collected and assessed by a pharmacist. Based on this assessment, on June 29, 1998, a report of collated practice patterns of the appropriateness of observed patterns of drug prescriptions was presented in the Pharmacy Department newsletter that was distributed to clinicians in the hospital.

#### Concurrent DUR hospital

On May 4, 1998, criteria were distributed to all prescribers and pharmacists in the concurrent DUR hospital. Between May 11 and August 14, 1998, at the end of each day, a designated pharmacist had to screen all cisapride prescriptions for appropriateness. When a prescription was deemed inappropriate, the pharmacist had to communicate directly with the physician to discuss the deviation from criteria and propose alternative solutions. Physicians were free to change their prescription. In February 1999, the pharmacy department distributed a newsletter to physicians and pharmacists. The aggregate results on the quality of cisapride prescriptions issued during the 3-month intervention period of May to August 1998 were presented in this newsletter.

### Data collection

We identified patient medical records using the computerized pharmacy database of each hospital. In the control hospital, we reviewed the records of patients who had been prescribed cisapride between November 1994 and September 1999. There was no intervention in the control hospital. In the retrospective DUR hospital, the period of observation was from October 1994 to February 1999 with the first intervention implemented at the 41^rst ^month while the period of observation was from June 1995 to September 1999 in the concurrent DUR hospital, with the first intervention being implemented at the 35^th ^month.

Periods of observation differed slightly among hospitals mainly because pharmacy computer systems had not been implemented at the same time in each hospital. Moreover, cisapride was de-listed in February 1999 from the hospital formulary of the retrospective DUR hospital. A medical archivist abstracted records of all patients who were prescribed cisapride in each hospital during each hospital study period. Information needed to apply DUR criteria (see Table 1) was abstracted. Data included patient age, gastro-intestinal diagnoses, symptoms and procedures with date of occurrence, contra-indicated drugs, cisapride doses, date and time of administration, international normalized ratios, and drugs interacting with cisapride. Data was entered directly on a portable computer using Epi-Info software. The archivist was not aware of the study objectives and of the DUR criteria. Appropriateness of use was later assessed using data collected by the archivist. Explicit criteria were applied by computer.

### Analyses

#### Appropriateness of use

The unit of analysis is the monthly aggregated prescriptions for all physicians in a given hospital. We divided the study period into equal observation periods of four weeks. For each period, we assessed the proportion of appropriate prescriptions according to each one of the four criteria. For each patient, the appropriateness of the cisapride prescription was assessed both at the first day of treatment (day 1) and three days later (day 4). This second day-4 assessment was performed to allow for the measurement of the effect of the intervention during the course of treatment in the concurrent intervention hospital.

We first estimated the overall average proportion of appropriateness for the entire study period. This allowed a general comparison of the percentage of appropriate prescriptions according to each one of the criteria on day 1 and day 4 of treatment. Since appropriateness of prescriptions was almost perfect as to dosage and drug interactions at both day 1 and day 4, we did not carry out any further analysis on these two criteria and concentrated on the remaining two criteria: indication for use and combination therapy.

#### Time series

To test the effect of each type of DURs on the appropriateness of cisapride prescriptions, we used ARIMA modeling techniques [9-11]. For each hospital, we constructed 4 series of at least 52 observation periods (one series for each of the two criteria applied at day 1 and one series for each of the two criteria applied at day 4).

#### ARIMA analysis

We estimated the ARIMA (autoregressive, seasonality, and moving average) coefficients for the four series in each of the three study hospitals. After obtaining a fitting model for each of these 12 series, we created indicator series for each experimental hospital. These latter series contained only 0s and 1s, 0s for all time periods prior to the introduction of the DUR in the hospital and 1 afterward. Since two separate activities correspond to the introduction of the DUR, the distribution of criteria and the diffusion of the results, two series were created in each experimental hospital. Four such series were created for the control hospital, one corresponding to each of the experimental series. Two coefficients were then introduced in the equations to model the interventions: t ω_0 _as an estimate of the average differences in the proportion of appropriate prescriptions (according to criteria) before and after the intervention, and δ_1 _to estimate whether the effect of the intervention is constant (δ_1 _= 0), expanding (δ_1 _> 0) or fading away (δ_1 _< 0).

In order to illustrate the evolution of the time series in a way that is more illuminating, we built graphical representations of the time series through a smoothing method. In order to attenuate the noise created by the random components in the raw data, we used Tukey's smoothing procedure [12]. This lag 3 smoothing technique consists in replacing the Ith value in the series with the mean of the i-1, i and i+1 values. For each series, we performed 3 mean smooths with a lag of 3. It should be noted that the purpose of the graphic representations is for ease of interpretation only, the ARIMA models were built using raw data, and the parameters of the models were used to draw the conclusions about the effect of the DUR on prescriptions appropriateness.

### Statistical analysis

We used t-tests to compare the ω_0 _coefficients of the experimental series with those of the control series. A significant t indicates a significant intervention effect over and above the secular trend captured by the variation in the control series. We conducted the analyses using the SAS software package [13].

## Results

For each hospital, the overall average proportion of appropriate prescriptions by criterion of appropriateness is displayed in Table 2. For the entire study period, appropriateness was almost perfect with regards to dosage and drug interactions at both day 1 and day 4 in all three hospitals. For the other two criteria, appropriateness seems to be improving between day 1 and day 4 in all hospitals including the control one. Overall, appropriateness was higher on indication for use than on combination therapy criterion.

**Figure 1:**
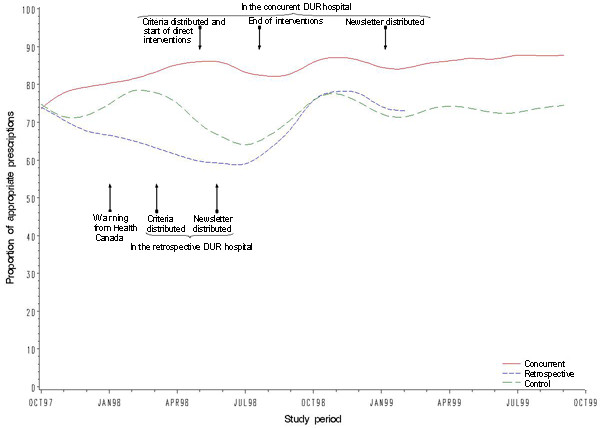
Proportion of cisapride prescriptions appropriate at day 1 in regards of indication for use in the concurrent DUR, retrospective DUR and control hospitals between October 1997 and October 1999.

**Figure 2:**
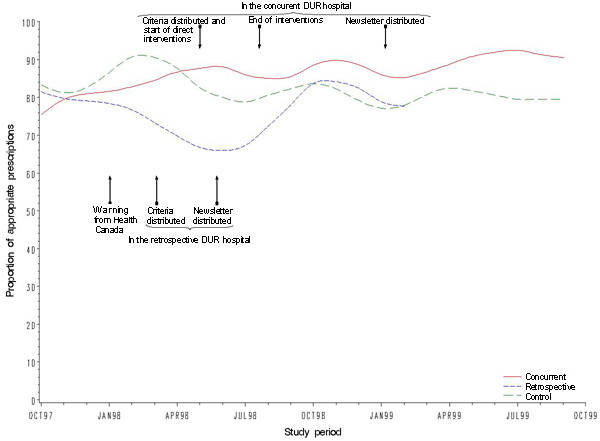
Proportion of cisapride prescriptions appropriate at day 4 in regards of indication for use in the concurrent DUR, retrospective DUR and control hospitals between October 1997 and October 1999.

Table 3 reports the effect of each DUR intervention on the appropriateness of prescriptions for the indication for use. Time-series results are also presented for the control hospital. Since all omega values but one are positive in both DUR experimental hospitals, it describes a general tendency for improvement whereas this tendency is toward reduced appropriateness in the control hospital in which omega values are all negative. In the concurrent DUR hospital, the improvement induced by the distribution of criteria and the start of pharmacist interventions is statistically significant for prescriptions at day 4, but not at day 1. Figures 1 and 2 depict these time series and the timing of the interventions. It shows that, during the direct interventions period conducted in the concurrent DUR hospital, the appropriateness of prescriptions for the indication for use has decreased in the control hospital. The graphical representations are concordant with the respective negative omega values reported in Table 3. In contrast, during this same period in the concurrent DUR hospital, the appropriateness of prescriptions has remained stable at day 1 and increased at day 4. Again, the graphical representations are concordant with the omega values reported in Table 3. At day 1, the omega value was positive although not statistically significant (P value of 0.11) whereas it was both positive and statistically significant when prescriptions were assessed after the initiation of treatment at day 4 (P value of 0.03). When these changes are compared with those in the control hospital (see Table 3), there is a statistically significant improvement at both day 1 (T = 2.52; P = 0.01) and day 4 (T = 2.62; P = 0.01) in the concurrent DUR hospital only. This improvement is associated with the distribution of criteria to physicians and pharmacists as part of the DUR program.

**Figure 3:**
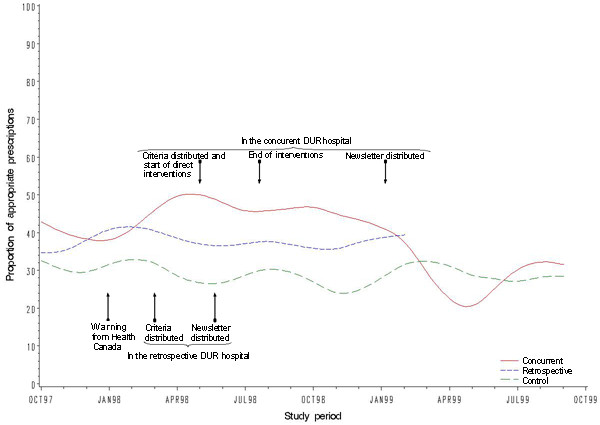
Proportion of cisapride prescriptions appropriate at day 1 in regards of combination therapy in the concurrent DUR, retrospective DUR and control hospitals between October 1997 and October 1999.

**Figure 4:**
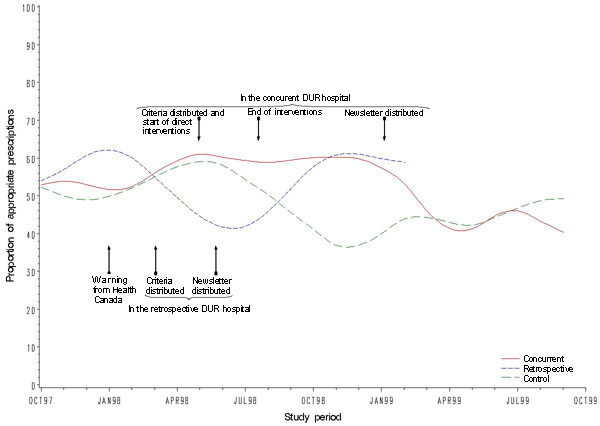
Proportion of cisapride prescriptions appropriate at day 4 in regards of combination therapy in the concurrent DUR, retrospective DUR and control hospitals between October 1997 and October 1999.

The effect of the DUR interventions on the appropriateness of combination therapy is presented in Table 4 and depicted in Figures 3 and 4. In all settings the appropriateness has decreased over the study period as illustrated by the fact that 15 of the 16 omega values are negative. Moreover, this decrease seems to be more pronounced in the concurrent DUR hospital in which it is statistically significant after the diffusion of collated results at both day 1 (ω_0 _= -16.72; P = 0.01) and 4 (ω_0 _= -14.29; P = 0.04). There is however no statistically significant difference between the control hospital and the two others.

## Discussion

Three important findings emerge from this evaluation. First, conducting a retrospective DUR program had no significant effect on prescription quality. Second, the concurrent DUR program is associated with a significant improvement of the appropriateness of prescriptions for the indication for use. Third, in all hospitals the appropriateness for combination therapy decreased over the study period.

Dissemination of the criteria and of collated results to clinicians were the only interventions in the retrospective DUR hospital. Therefore it may explain the lack of effectiveness of this approach. Although dissemination of criteria may predispose physicians to modify their prescribing behavior, it may not be sufficient. Previous studies have shown that guidelines dissemination alone has no significant effect on prescribing behavior [14] while studies assessing the impact of DUR intervention letters or reports on prescribing for patients not specifically identified in the letter or report cannot be found in the literature [6]. In theory, such report could reinforce the prescribing behavior change though our results suggest it is not sufficient.

In addition to the dissemination of criteria, the concurrent DUR interventions included direct communication between the pharmacist and the physician during the course of treatment, and the distribution of collated results. As indicated above, disseminating criteria may predispose physicians to behavior change while the other two activities may enable and reinforce the change, respectively. Our results suggest that as a package these three interventions may be sufficient to induce prescribing behavior change in physicians. This effect was however limited to the indication for use criterion.

The appropriateness of prescriptions with regards to combination therapy decreased in all hospitals over the study period. As the decrease in appropriateness was also observed in the control hospital, it suggests a strong secular trend that would have been interpreted wrongly as an adverse effect of the interventions had we conducted this study without an adequate control group. Secular trends in prescribing behavior have been observed in the past [15].

This overall decrease in appropriateness for the combination therapy criterion was not expected. As reports of adverse events have been published during the study period, it may have influenced physicians and pharmacists to focus more on the indication for use than on combination therapy. In July 1996, in a newsletter sent to physicians and pharmacists Health Canada informed that cisaprise was contraindicated in patients taking drugs that inhibited the cytochrome P450-3A4 enzymes that metabolize cisapride or in those taking drugs that could prolong the QT interval since those patients were at increased risk of developing severe ventricular arythmia or *torsades de pointe*. The list of all drugs – including cisapride- that could prolong the QT interval or *torsades de pointe *appeared in another newsletter sent to health professionals in January 1998. These warning newsletters may have had an effect on the prescribing behavior of physicians in our study although previous research has shown such letters alone had no effect [16]. In the USA, change in labeling, black-box warning and press release by the FDA, together with a "Dear Health Care Professional" letter sent by the manufacturer to physicians and pharmacists showed no effect in reducing prescribing of contraindicated drugs to patients on cisapride [17]. Although similar actions are not likely to have impact differently on Canadian physicians and pharmacists, the feedback messages on the combination therapy criterion in our study, if any, may thus have been perceived as clinically unimportant. Indeed, the concomitant prescription of cisapride with a proton pomp inhibitor although inappropriate is not dangerous. On the other hand, physicians seem to have considered very seriously the inappropriateness of prescribing concomitantly cisapride and contraindicated drugs, as illustrated by the very high proportion of prescriptions being appropriate on this criterion.

Our results also show that the appropriateness of prescriptions has improved in all settings and for both criteria between day 1 and day 4. It suggests that either physicians are reviewing their own prescriptions and/or pharmacists, as part of their usual practice, are intervening even in the absence of a formal DUR program [18]. This strong improvement effect illustrates the importance of a control group when one wants to assess the effect interventions have during the course of a treatment [14].

Our study has strengths and limitations. The greatest strength of this study is the use of a control-group time-series analysis design based on records of drug prescriptions for a period of over four years. We were therefore able to account for prior trends and for regression toward the mean i.e., the tendency for deviant prescriptions to approach the mean on subsequent observation [14]. Moreover, this study was conducted in the context of current clinical practice with no attempt from the investigators to impose the selection of the drug or to enhance compliance of pharmacist in delivering DUR interventions. As a limitation however, there was a lack of randomization. In addition, because no information on prescribing physicians was collected it was not possible to describe physician characteristics in each hospital and test their comparability. Another limitation lies in the fact that the unit of analysis is a hospital level aggregation of prescriptions. Although this insures the reliability of our observations of the proportion of appropriate prescriptions, their number was still relatively modest thus limiting the power to detect an intervention effect. Finally, the concurrent DUR interventions lasted only three months. The effect of this type of DUR could therefore have been greater had we assessed a program implemented on a longer term. It is however current practice in Quebec hospitals to conduct concurrent DUR programs of short duration, in general six weeks [19].

Care should be taken before generalizing the results of this study to all DUR programs as the effect of those programs depends heavily on the targeted drug, the interventions offered as part of the programs, and on the criterion against which appropriateness is assessed. There is also a need for further research to assess whether the effect of DUR interventions on the drug prescribing process impact on patient outcomes and on the efficiency of health care services.

The decrease in the appropriateness of prescriptions for combination therapy highlights the need to design or integrate more effective interventions. In this regard, as part of a concurrent DUR program, computer-based interventions look promising. Systems designed to warn pharmacists about the inappropriate prescription of a drug have been shown to contribute at improving ambulatory prescribing patterns for the elderly patients [20]. Computer physician order entry displaying drug utilization recommendations could also be integrated in DUR programs [21]. There is also increasing evidence that providing physicians with prescriber-specific and patient-specific profiles positively alter the prescribing behavior of physicians in the ambulatory setting [22-24]. To achieve greater impact, all the preceding interventions could be offered in concert with other well established interventions such as academic detailing [25]. Further research is needed to identify the mix of interventions that would be the most cost-effective.

## Conclusion

In conclusion, we have conducted this study in the routine clinical practice setting with no intervention from us in the clinical process. Although cisapride has since been withdrawn from the Canadian market, this study is relevant as it shows that a retrospective DUR the way it is generally conducted in Canadian hospitals may not be sufficient to improve the quality of prescribing. On the other hand, a concurrent DUR with direct feedback to prescribers seems effective to improve the appropriateness with regard to the indication for use. Nevertheless, it may have negative effects on other component of the quality of the prescriptions. Since the effect of DURs varies with both the type of interventions conducted and the criterion applied, there is a need for further research in other settings and with other drugs.

## Competing interests

The author(s) declare that they have no competing interests.

## Authors' contributions

Jean-Pierre Grégoire developed the design, and led the statistical analyses and the writing of the manuscript. Jocelyne Moisan, Louise Potvin, Isabelle Chabot, René Verreault and Alain Milot all contributed to the design, the statistical analysis and the writing of the paper.

## Pre-publication history

The pre-publication history for this paper can be accessed here:



## Figures and Tables

**Table 1:** Criteria for the appropriate use of Cisapride

**1. Indication for use**	
**Criteria**	**Exception**
May be used:	May be used
1.1 For empirical treatment (no investigation): symptoms of dysmotility with or without gastro-oesophagus reflux symptoms	1.1 1) If dysphagia, more than 3 vomiting episodes per week, or gastro-intestinal bleeding not related to another cause 2) If mechanical obstruction or intestinal perforation.
1.2 For non empirical treatment (with investigation): symptoms of dysmotility with or without gastro-oesophagus reflux symptoms AND absence of moderate to severe oesophagitis at endoscopy (Savary-Miller grade III or IV)	1.2 1) For moderate to severe oesophagitis if prescribed with a proton pump inhibitor (lansoprazole, omeprazole, pantoprazole) 2) If mechanical obstruction or intestinal perforation.
1.3 For gastroparesia	1.3 If mechanical obstruction or intestinal perforation.
1.4 For chronic pseudo-obstruction	1.4 If mechanical obstruction or intestinal perforation.
	
**2. Contra-indicated drugs (combination therapy)**	
**Criteria**	**Exception**
Must not be used:	May be used:
2.1 With domperidone or metoclopramide	
2.2 With proton pump inhibitors	2.2 With proton pump inhibitors when moderate to severe oesophagitis
2.3 With an Anti-H2	
	
**3. Dosage**	
**Criterion**	**Exception**
3.1 Maximum of 80 mg/day; 20 mg/dose, 4 doses/day	3.1 If > 80 years old or hepatic failure: Maximum of 40 mg/day; 10 mg/dose
	
**4. Drug interactions**	
**Criterion**	**Exception**
Must not be used: 4.1 Concurrently with erythromycin, clarithromycin, fluconazol, ketoconazol, itraconazol, nefazodone, nicoumalone or warfarin	May be used: 4.1 In presence of nicoumalone or warfarin if international normalized ratio obtained 1 week after addition of cisapride and at cessation if stopped

**Table 2:** Average proportion of appropriate prescriptions on day 1 and day 4 of treatment during the observation period by hospital and by criterion of appropriateness

			(95% Confidence Interval)			
**Criteria**	**DUR hospital**	**Number of prescriptions**	**Day 1 of treatment**		**Day 4 of treatment**	

Indication for use	concurrent†	771	82.27	(78.48 – 85.70)	84.51	(81.29 – 87.73)
						
	retrospective‡	692	65.54	(61.62 – 69.46)	76.04	(72.65 – 79.43)
	control§	1 040	76.77	(73.53 – 80.01)	83.20	(80.58 – 85.82)
						
Combination therapy	concurrent†	771	45.20	(40.95 – 49.45)	55.26	(51.52 – 59.00)
	retrospective‡	692	39.27	(35.64 – 42.90)	53.68	(49.54 – 57.82)
	control§	1 040	37.87	(33.86 – 41.88)	52.75	(49.40 – 56.10)
						
Dosage (not further analysed)	concurrent†	771	95.81	(94.53 – 97.09)	96.35	(95.16 – 97.54)
	retrospective‡	692	96.33	(94.94 – 97.72)	98.16	(97.03 – 99.29)
	control§	1 040	96.72	(95.59 – 97.85)	97.24	(96.20 – 98.28)
						
Drug Interactions (not further analysed)	concurrent†	771	98.00	(96.99 – 99.01)	98.41	(97.45 – 99.37)
	retrospective‡	692	96.97	(95.75 – 98.19)	96.88	(95.67 – 98.09)
	control§	1 040	97.34	(96.43 – 98.25)	98.37	(97,59 – 99.15)

^† ^based on 52 months of observation

^‡ ^based on 53 months of observation

^§ ^based on 57 months of observation

**Table 3:** Effects of the DURs on the appropriateness of prescriptions by type of interventions: indication for use

			**DUR experiment** **hospitals**			**No DUR** ** (control hospital)**			**Difference between** ** DUR – no DUR**	
**Type of DUR**	**Day of the evaluation**	**DUR intervention**	ω_0_	**(SE)**	**P-value**	ω_0_	**(SE)**	**P-value**	**T**	**P-value**

										
Retrospective	Day 1	Criteria distribution	1.11	(4.87)	0.82	-6.36	(3.46)	0.07	1.25	0.21
		Results diffusion	6.41	(5.62)	0.25	-7.02	(3.55)	0.05	1.71	0.09
	Day 4	Criteria distribution	-2.65	(4.20)	0.53	-2.46	(2.86)	0.39	0.04	0.97
		Results diffusion	3.29	(4.91)	0.50	-3.04	(3.06)	0.32	1.09	0.28
										
Concurrent	Day 1	Criteria distribution* and one-on-one interventions	5.90	(3.71)	0.11	-7.02	(3.55)	0.05	2.52	0.01
		Results diffusion†	4.66	(4.90)	0.34	-5.17	(3.75)	0.17	1.30	0.20
	Day 4	Criteria distribution* and one-on-one interventions	12.70‡	(5.71)	0.03	-4.08	(2.92)	0.16	2.62	0.01
		Results diffusion	5.47	(4.58)	0.23	-2.98	(3.89)	0.44	1.41	0.16

* In the concurrent DUR, distribution of criteria corresponds to the start of the direct interventions 3-month period.

† Results were distributed in the form of a newsletter

‡ δ1 = -0.83 (SE = 0.25) P-value = 0.001

**Table 4:** Effects of the DURs on the appropriateness of prescriptions by type of interventions: combination therapy

			**DUR experiment** ** hospitals**			**No DUR** ** (control hospital)**			**Difference between** ** DUR – no DUR**	
**Type of DUR**	**Day of the evaluation**	**DUR intervention**	ω_0_	**(SE)**	**P-value**	ω_0_	**(SE)**	**P-value**	**T**	**P-value**

										
Retrospective	Day 1	Criteria distribution	-3.46	(4.48)	0.44	-11.56	(8.96)	0.20	0.81	0.42
		Results diffusion	-2.14	(5.26)	0.68	-5.93	(9.33)	0.53	0.35	0.73
	Day 4	Criteria distribution	-2.98	(5.12)	0.56	-7.58	(4.22)	0.07	0.69	0.49
		Results diffusion	1.08	(6.01)	0.86	-11.73	(4.20)	0.01	1.75	0.08
										
Concurrent	Day 1	Criteria distribution* and one-on-one interventions	-10.75	(5.74)	0.06	-10.04	(8.93)	0.26	-0.07	0.94
		Results diffusion†	-16.72	(6.45)	0.01	-2.68	(7.59)	0.72	-1.41	0.16
	Day 4	Criteria distribution* and one-on-one interventions	-4.71	(5.59)	0.40	-7.69	(4.32)	0.08	0.42	0.81
		Results diffusion	-14.29	(6.80)	0.04	-5.85	(5.87)	0.32	-0.94	0.35

* In the concurrent DUR, distribution of criteria corresponds to the start of the direct interventions 3-month period.

† Results were distributed in the form of a newsletter
